# A comparison between the adductor pollicis muscle and the abductor digiti minimi muscle using electromyography AF-201P in rocuronium-induced neuromuscular block: a prospective comparative study

**DOI:** 10.1186/s12871-022-01656-y

**Published:** 2022-04-23

**Authors:** Hajime Iwasaki, Hanae Sato, Shunichi Takagi, Osamu Kitajima, Sarah Kyuragi Luthe, Takahiro Suzuki

**Affiliations:** 1 Department of Anesthesiology, Nihon University School of Medicine, 30-1, Oyaguchi Kamicho, Itabashi-Ku, Tokyo, 173-8610 Japan; 2 Department of Anesthesia, Indiana University School of Medicine, 1130 W. Michigan St, Fesler Hall 204, Indianapolis, IN 46202 USA

**Keywords:** Abductor digiti minimi muscle, Adductor pollicis muscle, Electromyography, Neuromuscular monitoring, Rocuronium, Sugammadex

## Abstract

**Background:**

The AF-201P, a new electromyography (EMG)-based neuromuscular monitor has been developed recently. The aim of this clinical study was to compare two ulnar nerve innervated muscles: the adductor pollicis (AP) muscle and the abductor digiti minimi (ADM) muscle during the recovery from rocuronium-induced neuromuscular block by using EMG AF-201P.

**Methods:**

Twenty patients undergoing surgery with general anesthesia were enrolled in the study. During total intravenous general anesthesia, train-of-four (TOF) and post-tetanic counts (PTC) responses following 0.9 mg/kg rocuronium administration were concurrently monitored at the AP and the ADM muscles with EMG AF-201P on the opposite arms. At the end of the surgery, sugammadex 2 mg/kg was administered when TOF counts of 2 (TOFC2) was observed at both muscles. The primary outcome of the study was time from administration of rocuronium to first appearance of PTC response (first PTC). The secondary outcomes of the study were time from administration of rocuronium to TOF count of 1 (TOFC1), time from first PTC to TOFC1 (PTC-TOF time), time to TOFC2, and time from administration of sugammadex to TOF ratio ≥ 0.9. Agreement between the two muscles was assessed using the Bland–Altman analysis. Data are expressed as mean ± standard deviation.

**Results:**

Nineteen patients were included in the analysis. Time to first PTC was significantly faster at the ADM muscle than the AP muscle (24.4 ± 11.4 min vs 32.4 ± 13.1 min, *p* = 0.006). PTC-TOF time was significantly longer with the ADM muscle than the AP muscle (19.4 ± 7.3 min vs 12.4 ± 10.6 min, *p* = 0.019). There were no significant differences in time to TOFC2 and sugammadex-facilitated recovery between the two muscles. Bland–Altman analyses showed acceptable ranges of bias and limits of agreement of the two muscles.

**Conclusions:**

The ADM muscle showed a good agreement with the AP muscle during rocuronium-induced neuromuscular block but faster recovery of PTC response when using EMG.

**Trial registration:**

UMIN-CTR (Registration No. UMIN000044904). Registered 19 July 2021 -Retrospectively registered, https://center6.umin.ac.jp/cgi-bin/ctr_e/ctr_view.cgi?recptno=R000051290.

## Background

Quantitative neuromuscular monitoring is known to be the most accurate method to detect postoperative residual neuromuscular block [1, 2]. Therefore, routine use of quantitative neuromuscular monitoring is recommended whenever neuromuscular blocking agents are used in clinical anesthesia. Although the most frequently used monitor was acceleromyography (AMG), the AF-201P (Nihon-Kohden, Tokyo, Japan), a new electromyography (EMG)-based neuromuscular monitor module which connects to specific display unit (VA-201R, Nihon-Kohden, Tokyo, Japan) or to compatible patient monitor has finally been developed recently. It evokes and measures the muscle compound action potentials by a single-use stimulating and sensing electrode. Recent literature showed a good correlation and agreement between the AF-201P and AMG in evaluating the depth of deep neuromuscular block [3]. Both the adductor pollicis (AP) muscle and the abductor digiti minimi (ADM) muscle for ulnar nerve stimulation are recommended for detecting stable compound potentials when using EMG. However, little is known about the clinical differences in the responses to neuromuscular blocking agents between the AP muscle and the ADM muscle during neuromuscular monitoring with EMG.

The aim of this clinical study was to compare the responses of the two most commonly-employed muscle with ulnar nerve stimulation using EMG: the AP muscle and the ADM muscle during the recovery from rocuronium-induced neuromuscular block.

## Methods

Ethical approval for this study (RK-210209–02) was provided by Nihon University Itabashi Hospital Clinical Research Ethics Committee, Tokyo, Japan on 19 February 2021. This trial was registered in the University Hospital Medical Information Network under registration number UMIN000044904 (19/07/2021). After receiving written informed consent, twenty patients aged ≥ 20 years old undergoing surgery with general anesthesia were enrolled in the study. We excluded patients with an American Society of Anesthesiologists physical status classification ≥ IV, patients with a history of allergic reaction to neuromuscular blocking agents, patients with hepatic disease, patients with neuromuscular disease, and patients who were receiving medication known to interfere with neuromuscular function. On arrival to the operating room, all patients were monitored by an electrocardiogram, noninvasive blood pressure, and pulse oximetry. An intravenous catheter was inserted into the forearm or dorsal vein. After preoxygenation, general anesthesia was induced with 1–2 μg/kg fentanyl, 3–4 μg/ml target-controlled infusion of propofol, and 0.1–0.3 μg/kg/min remifentanil. After endotracheal intubation, anesthesia was maintained with 2–4 μg/ml target-controlled infusion of propofol and 0.1–0.3 μg/kg^/^min remifentanil, targeting a bispectral index of 40–50. An upper body forced-air warming device was used throughout the surgery to keep central skin temperature ≥ 35℃. End-tidal CO_2_ was maintained at 35–40 mmHg.

### Neuromuscular management

**Fig. 1:**
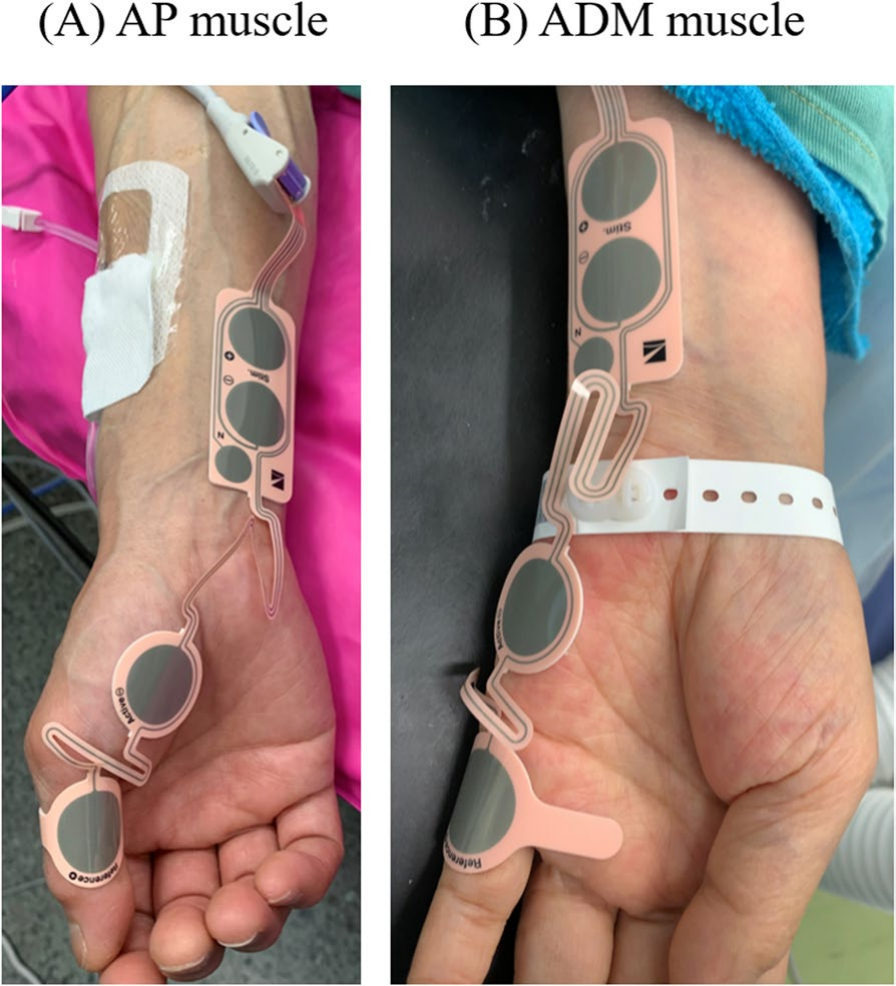
Set up of the electromyography (AF-201P, Nihon-Kohden, Tokyo, Japan). **A** Monitoring the abductor pollicis (AP) muscle. **B** Monitoring the abductor digiti minimi (ADM) muscle

Prior to induction of anesthesia, EMG AF-201P (Nihon-Kohden, Tokyo, Japan) were applied to the AP muscle and the ADM muscle of the different arms. After proper skin preparation, each single-use surface electrode (NM-345Y, Nihon-Kohden, Tokyo, Japan) was placed over the AP muscle (Fig. 1A), and the ADM muscle of the opposite arm (Fig. 1B). After induction of anesthesia, train-of-four (TOF) stimulation was delivered at 2 Hz for 1.5 s every 15 s following automated calibration of the supramaximal current and the responses. After confirmation of stable baseline TOF responses, all the patients received rocuronium 0.9 mg/kg intravenously. Post-tetanic count (PTC) stimulations were performed every 6 min during TOF count of 0. PTC stimulation was discontinued after the first detection of the PTC response to prevent facilitation of the TOF recovery [4]. Subsequently, we observed the spontaneous recovery of rocuronium-induced neuromuscular block by waiting until three continuous TOF counts of 2 (TOFC2) were observed in both monitoring sites. Additional doses of rocuronium 0.1–0.2 mg/kg were administered to maintain TOF counts ≤ 2 when necessary. At the end of surgery, sugammadex 2 mg/kg was administered when three continuous TOFC2 were observed in the both muscles.

### Outcomes of the study

The primary outcome of the study was the time (minutes) from administration of rocuronium to first appearance of PTC response (first PTC). The secondary outcomes of the study were supramaximal stimulation current (mA), time (seconds) from administration of rocuronium to TOF count of 0 (onset time), time (minutes) from administration of rocuronium to first reappearance of TOF count of 1 (TOFC1) (time to TOFC1), time (minutes) from first appearance of PTC to first reappearance of TOFC1 (PTC-TOF time), time (minutes) from administration of rocuronium to first reappearance of TOFC2 (time to TOFC2), and time (seconds) from administration of sugammadex to TOF ratio ≥ 0.9 (recovery time).

### Sample size and statistical analysis

A previous study revealed that the average ± standard deviation (SD) time for first appearance of PTC following 1.0 mg/kg rocuronium administration was 37.7 ± 12.3 min [5]. We considered a 30% difference in the recovery time observed between the two muscles to be clinically relevant. To detect the difference with an α value of 0.05 and a power of 0.8, 19 patients were required to be included in this study. We determined our sample size of 20 patients for any anticipated drop outs. Results are expressed as mean ± SD.

We used paired t-test to analyze the differences of the outcomes between the two monitoring sites. To assess the agreement between the two monitoring sites, Bland–Altman analysis and the biases and limits of agreement for each variable were calculated. All statistical analyses were performed using GraphPad Prism® version 7.03 (GraphPad Software, Inc., La Jolla, CA) and a *P*-value of < 0.05 was considered statistically significant.

## Results

Twenty subjects (aged 23–77 years) were enrolled in this study between April and August 2021. One patient was excluded from the analysis given that the TOF responses did not disappear following initial administration of rocuronium. Patients’ characteristics of age, weight, body mass index, number of female/male were 44.3 ± 16.1 y, 59.4 ± 13.7 kg, 22.6 ± 4.1 kg/m^2^, and 4/15, respectively.

Results of primary and secondary outcomes of the study are shown in Table 1. There was no significant difference in the supramaximal current and onset time between the muscles. Time to first PTC was significantly faster with the ADM muscle than the AP muscle. Whereas, there was no significant difference in the time to TOFC1 between the two muscles, and therefore, the PTC-TOF time was significantly longer with the ADM muscle than the AP muscle. There was no significant difference in time to TOFC2, and time to TOF ratio ≥ 0.9 between the AP muscle and the ADM muscle. The ADM muscle showed TOF counts of 3 or 4 while the AP muscle showed TOFC2 in 47% (9/19) of the patients. The ADM muscle and the AP muscle simultaneously showed TOFC2 in 32% (6/19) of the patients. The AP muscle showed TOF counts 3 or 4 when the ADM muscle showed TOFC2 in 21% (4/19) of the patients.

**Table 1:** Results of primary and secondary outcomes of the study

	AP muscle	ADM muscle	P value
Supramaximal current (mA)	41.1 ± 11.8	38.0 ± 13.2	0.34
Onset time (seconds)	92.4 ± 41.3	93.0 ± 27.2	0.96
Time to first PTC (minutes)	32.4 ± 13.1	24.4 ± 11.4	0.006
Time to TOF count of 1 (minutes)	44.9 ± 13.6	43.9 ± 13.1	0.72
PTC-TOF time (minutes)	12.4 ± 10.6	19.4 ± 7.3	0.019
Time to TOF counts of 2 (minutes)	55.8 ± 14.4	53.8 ± 15.1	0.45
Time to TOF ratio ≥ 0.9 (seconds)	83.4 ± 35.3	88.1 ± 44.8	0.73

Results are expressed as mean ± standard deviation

*AP* Adductor pollicis, *ADM* Abductor digiti minimi, *TOF* Train-of-four, *PTC* post-tetanic count

**Fig. 2:**
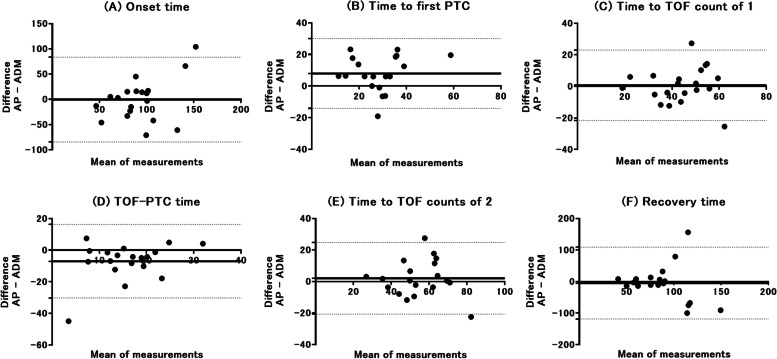
Bland–Altman plot illustrating the differences of **A** onset time, **B** time to first post-tetanic count (PTC) response, **C** time to train-of-four (TOF) count of 1, **D** PTC-TOF time, **E** time to TOF counts of 2, and **F** recovery time between the adductor pollicis (AP) muscle and the abductor digiti minimi (ADM) muscle as a function of the two measurements. Onset time is defined as time from rocuronium administration to TOF count of 0. Time to first PTC is defined as time from rocuronium administration to first appearance of PTC response. Time to TOF count of 1 is defined as time from rocuronium administration to first reappearance of TOF count of 1. PTC-TOF time is defined as time from first appearance of PTC to first reappearance of TOF count of 1. Time to TOF counts of 2 is defined as time from rocuronium administration to TOF counts of 2. Recovery time is defined as the time from sugammadex administration to TOF ratio ≥ 0.9. Solid horizontal line represents the bias and dotted horizontal lines represent the upper and lower limits of agreement with 95% confidence intervals

As shown in Fig. 2, Bland–Altman analyses showed acceptable ranges of bias (difference calculated by the AP muscle subtracted by the ADM muscle) and limits of agreement of the two monitoring sites. The biases and limits of agreement [95% confidence interval] were -0.53 ± 42.8 [-84.5 to 83.4] for the onset, 8.0 ± 11.3 [-14.1 to 30.1] for the time to first PTC, 0.73 ± 11.4 [-21.6 to 23.0] for the time to TOFC1, -7.0 ± 11.9 [-30.3 to 16.2] for the PTC-TOF time, 2.0 ± 11.6 [-20.7 to 24.8] for the time to TOFC2, and -4.7 ± 58.1 [-118.5 to 109.2] for recovery, respectively.

## Discussion

This comparative study found that time from administration of rocuronium to first appearance of PTC response was significantly faster with the ADM muscle than the AP muscle when using EMG AF-201P. In contrast, PTC-TOF time was significantly longer with the ADM muscle than the AP muscle. There were no differences in time to TOFC1 and time to TOFC2 between two monitoring sites.

Respiratory muscle such as diaphragm is known to be resistant to neuromuscular blocking agents referred to as respiratory sparing effect [6]. Similar to the diaphragm, the ADM muscle contains higher ratio of fast-twitch muscle (type II fiber) compared with the AP muscle [7]. Therefore, we first hypothesized that the ADM muscle would show faster recovery of both PTC and TOF responses than the AP muscle. However, although the time to first PTC was significantly faster in the ADM muscle than the AP muscle, no differences were observed in time to recovery of TOF responses between the two muscles in our study. Therefore, results of our study may not be simply explained by the differences in sensitivity of the two muscles to neuromuscular blocking agents. According to the previous reports, contractions of the fast-twitch muscles are potentiated following tetanus stimulation while slow-twitch muscles are depressed [8, 9]. In our study, given that the ADM muscle contains more fast-twitch muscles than the AP muscle, the ADM muscles may have shown a marked post-tetanic potentiation and a significant faster recovery of the PTC.

The biases and limits of agreement in Bland–Altman plots indicated that the data observed by both monitoring sites presented good agreement in our study. A previous study has compared three ulnar nerve innervated muscles, the AP muscles, the ADM muscles, and the first dorsal interosseous (FDI) muscles by using a different EMG-based neuromuscular monitor (Datex-Ohmeda GE Healthcare Neuromuscular Transmission Monitor-EMG, Helsinki, Finland) [10]. Accordingly, although the ADM muscle was the most resistant to neuromuscular block when compared with the AP muscle and the FDI muscle, the differences in recovery of neuromuscular function between the muscles during shallow neuromuscular block were small [10]. Our study corroborates this previous study and supports the agreement between the AP muscle and the ADM muscle during moderate neuromuscular block as well.

The recovery time to TOF ratio ≥ 0.9 following administration of 2 mg/kg (the recommended dose) sugammadex when TOFC 2 (moderate neuromuscular block) is reported to be within 3 min [11]. The results of our study support the efficacy of using sugammadex under the guidance of the AP muscle and the ADM muscle with EMG as the mean recovery times were approximately 1.4 min and 1.5 min, respectively, from sugammadex administration when the AP muscle and the ADM muscle showed TOFC2 to TOF ratio ≥ 0.9. As the ADM muscle recovered faster than the AP muscle during deep neuromuscular block in our study, further investigation is required to confirm the recovery time from administration of the recommended dose of sugammadex during deep neuromuscular block (PTC of 1 or 2).

Our study has several limitations. First, two separate monitors were applied to the same subject but on opposing arms. However, according to the previous reports, the location of the devices (ipsilateral of contralateral) and hand dominance did not affect the measurements of neuromuscular block [12–14]. Second, since sugammadex was administered when both monitors showed TOFC2, spontaneous recovery from TOFC2 to complete recovery was not assessed between the two monitoring sites. Further investigation is required to confirm the agreement between the AP muscle and the ADM muscle during shallow and minimal neuromuscular block. Third, PTC stimulation was discontinued after the first detection of the PTC response to prevent facilitation of the TOF recovery. Therefore, we were unable to obtain the predicted curve of relationship between PTCs and time to TOFC1. According to our primary outcome, the predicted curve of PTCs and the time to TOFC1 may be different between the AP muscle with the ADM muscle. Further studies are required to investigate the relationship of PTCs and TOF responses during deep neuromuscular block with the ADM muscle. Forth, 3 elderly (> 65 years old) patients were included in the analysis. It seems that difference in time to first PTC between the AP muscle and the ADM muscle was larger with elderly patients than younger patients in our study (data not shown). As the number of elderly patients are limited in this study, further studies are required to confirm whether the difference between the AP muscle and the ADM muscle increases with elderly patients.

## Conclusions

The ADM muscle showed good agreement with the AP muscle during rocuronium-induced neuromuscular block but faster recovery of PTC response when using EMG AF-201P in this study. Our results suggest that monitoring both the AP muscle and the ADM muscle with EMG can be an indicator to decide the adequate dose of sugammadex to reverse moderate neuromuscular block and to confirm adequate recovery of neuromuscular function.

## Data Availability

The datasets used and analyzed during the current study are available from the corresponding author on request.

## Abbreviations

AMG: Acceleromyography
EMG: Electromyography
AP: Adductor pollicis
ADM: Abductor digiti minimi
TOF: Train-of-four
PTC: Post-tetanic count
SD: Standard deviation
